# Cross-Presenting XCR1^+^ Dendritic Cells as Targets for Cancer Immunotherapy

**DOI:** 10.3390/cells9030565

**Published:** 2020-02-28

**Authors:** Katherine M. Audsley, Alison M. McDonnell, Jason Waithman

**Affiliations:** 1 Telethon Kids Institute, University of Western Australia, Perth Children’s Hospital, Nedlands, WA 6009, Australia; 2 School of Biomedical Sciences, The University of Western Australia, Crawley, WA 6009, Australia; 3 National Centre for Asbestos Related Diseases, The University of Western Australia, QEII Medical Centre, Nedlands, WA 6009, Australia

**Keywords:** cross-presenting dendritic cells, DC-based therapy, immunotherapy, cancer

## Abstract

The use of dendritic cells (DCs) to generate effective anti-tumor T cell immunity has garnered much attention over the last thirty-plus years. Despite this, limited clinical benefit has been demonstrated thus far. There has been a revival of interest in DC-based treatment strategies following the remarkable patient responses observed with novel checkpoint blockade therapies, due to the potential for synergistic treatment. Cross-presenting DCs are recognized for their ability to prime CD8^+^ T cell responses to directly induce tumor death. Consequently, they are an attractive target for next-generation DC-based strategies. In this review, we define the universal classification system for cross-presenting DCs, and the vital role of this subset in mediating anti-tumor immunity. Furthermore, we will detail methods of targeting these DCs both ex vivo and in vivo to boost their function and drive effective anti-tumor responses.

## 1. Introduction

The revolutionary success of cancer immunotherapies harnessing T cell immunity has renewed interest in novel therapeutic strategies targeting dendritic cells (DCs). One of the most successful immunotherapy strategies in routine clinical use is immune checkpoint blockade therapy (ICB), which blocks inhibitory signaling pathways to activate tumor-specific T cells that would otherwise remain suppressed [1]. However, the majority of patients receiving ICB ultimately succumb to their disease, with therapy failure partially attributed to insufficient recruitment of tumor-specific T cells [2]. This highlights the need for effective vaccines targeting the generation of robust T cell immunity capable of synergizing with established treatments.

Since their discovery in 1973 [3], DCs have been recognized for their unique ability to link the innate and adaptive arms of the immune system via presentation of antigen to T cells. Consequently, they have long been considered attractive targets for anti-cancer therapies. There have been over 200 clinical trials evaluating the use of DC vaccines against cancer, whereby DCs are loaded ex vivo with cancer-derived antigens to induce T cell immunity [4,5]. Despite the success of Sipuleucel-T as an established treatment for prostate cancer [6], successful immunotherapies based on the concept of specifically targeting DCs for therapeutic benefit remain limited. In recent years, our increased knowledge of basic DC biology has led to the development of many new and novel DC-based strategies capable of promoting durable responses in cancer patients.

DCs are functionally heterogeneous and can broadly be categorized into three subsets. Plasmacytoid DCs (pDCs) are predominantly involved in anti-viral immunity and promoting tolerance to both innocuous- and self-antigens [7,8]. The conventional DCs (cDCs) consist of cDC1 and cDC2 subsets that are responsible for antigen presentation to CD8^+^ and CD4^+^ T cells in the context of MHCI and MHCII, respectively [9]. Finally, inflammatory DCs differentiate from monocytes during conditions of inflammation in the body, such as infection and cancer [10,11]. One of the potential reasons underlying the failure of early DC vaccination protocols was the use of monocyte-derived DCs, later realized to have a relatively poor antigen presentation capacity [5,10]. Current vaccination strategies take into consideration the increased antigen presentation capabilities and functional specialization of specific DC subsets. The cDC1 population is recognized for its unique capacity to cross-present exogenous antigen to CD8^+^ T cells, and is, therefore, a logical choice to induce effective cytotoxic T lymphocyte (CTL) responses with DC vaccination [4]. One of the issues confounding the targeting of cross-presenting DCs in the treatment of disease for many years was the lack of a classification system that encompasses this functional subset. In particular, while there was evidence for a functional counterpart in humans, the lack of a universal marker made translation of studies into humans difficult. Eventually, the discovery of a shared ontogeny for Batf3 [12,13,14] united the cross-presenting population, further supported by the identification of a universal surface marker on cross-presenting DCs—the chemokine receptor, XCR1 [15,16,17].

There is significant evidence for the role of cross-presenting DCs in cancer [13,18,19,20,21,22,23,24,25,26,27]. Focus is now being directed towards enhancing the function of these DCs, including improved antigen loading, proliferation, maturation, antigen presentation and recruitment in vivo. Current strategies include the use of adjuvants to promote maturation [23,28], chemokines to promote DC-CD8^+^ T cell interaction and migration [26,29,30], and antibody and chemokine constructs that target antigen to XCR1^+^ DCs [31,32,33]. Here, we will discuss the defining features of the cross-presenting DC population, methods of targeting them for the generation of effective CD8^+^ T cell-driven anti-tumor responses, and the potential for these approaches to synergize with ICB.

## 2. Cross-Presenting Dendritic Cells—A Functional Niche

Cross-presentation, first reported by Bevan and colleagues in the mid-1970′s, defines the process of internalizing exogenous antigen and shunting it into the MHC class I pathway for presentation to CD8^+^ T cells [34,35]. It is now well established that DCs are the major cross-presenting population [36] and play a critical role in the generation of viral and tumor-specific CTL responses [18,37,38]. Seminal work in mice by Shortman and colleagues identified cDC1 (CD11b^neg^) CD8-expressing DCs in secondary lymphoid organs as the cross-presenting subset [9,13,18,39,40,41,42]. Subsequently, it was determined that a proportion of migratory DCs could also cross-present, whereby CD103^+^CD11b^neg^ DCs excel in the cross-presentation of antigen from the lung [43,44,45], intestine [46,47] and skin [21,48,49]. A functional counterpart in humans was first defined as BDCA-3^+^ (CD141^+^) based on phenotypic and transcriptomic studies [17,50,51,52,53]. BDCA-3^high^ DC appear to represent the migratory equivalent in humans, a subset that was challenging to identify for many years due to the difficulty in obtaining sufficient samples [54,55,56]. In recent years, cross-presenting DCs in mice and humans have been unified by the identification of shared ontogeny and phenotypic markers (summarized in Table 1). cDC1s depend on the transcription factors IRF-8, Id2, and Batf3 for their development [12,13,14,57,58,59,60], and selectively express the C type lectin receptor CLEC9A (DNGR-1) [31,45,61] and the chemokine receptor XCR1 [15,16,59]. The exclusivity of XCR1 expression for cross-presenting DCs was confirmed with the development of an XCR1-specific antibody and generation of an XCR1-LacZ reporter mouse [15,16,59]. In line with this, there is a perfect correlation between Batf3 dependency and XCR1 expression, with all XCR1^+^ cells absent in Batf3^−/−^ mice [16,62]. Importantly, XCR1 is expressed on both lymphoid and peripheral cross-presenting DC in both mice and humans [16,62,63]. In addition, cDC1 share expression of the pattern recognition receptor (PRR) TLR3 [10,64], producing high amounts of IL-12 upon activation [65]. They are also known to be the major producers of IFN-λ following TLR3 ligation [66].

Both lymphoid-resident and migratory cross-presenting DCs are specialized in the uptake, processing and cross-presentation of antigen derived from stressed cells [59,67]. Originally, the superior cross-presentation ability of these DC was thought to be due to an exclusive ability to internalize exogenous antigen from apoptotic and necrotic cells [68,69]. One of the characteristic features of cross-presenting DC is, after all, the expression of surface receptors specific for dead and dying cell antigens, such as CLEC9A and CD36 [31,45,61,70]. However, studies in mice have shown that CD8^+^ and CD8^neg^ DCs can similarly capture both soluble and cell-associated antigens, as demonstrated by the ability of CD8^neg^ DCs to generate robust antigen-specific CD4^+^ T cell responses [39,40,41]. Only CD8^+^ DCs can present antigen on MHC class I complexes, however, indicating the existence of specialized intracellular processing machinery [40,41,69]. The exact mechanisms of intracellular processing remain elusive, although a preference for the cytosolic pathway has been demonstrated in both lymphoid-resident and migratory cross-presenting DC [71,72]. Unlike the vacuolar pathway, whereby antigen is degraded into peptides within the phagosome, the cytosolic pathway requires phagocytosed antigen to be exported into the cytosol for processing by the proteasome prior to MHC class I loading (reviewed extensively in [73,74]). Differential upregulation of proteins involved in MHCI and MHCII processing pathways by CD8^+^ and CD8^neg^ DC, respectively, could also explain the preferential processing of exogenous antigen for MHCI presentation [39]. XCR1 expression by cross-presenting DCs may also play a role, as it is known that XCR1 engagement by its ligand, XCL1, promotes interaction between cross-presenting DC and CD8^+^ T cells to enhance cross-priming [15,63]. With the identification of a universal surface marker, XCR1, to demarcate the Batf3-dependent cross-presenting DC lineage, we are now acutely primed to translate the use of these cells into the clinic.

## 3. Critical Role for Cross-Presenting DCs in Cancer

An essential role for DCs in cancer immune surveillance is well established [5,75]. A particular focus on the importance of XCR1^+^ DCs is warranted, due to their ability to orchestrate productive CD8^+^ T cell immunity. Here, we discuss the role of this population in mediating anti-tumor responses, including substantial evidence that demonstrates that their suppression within the tumor microenvironment (TME) impedes tumor clearance.

### 3.1. XCR1^+^ DCs and Cancer

As the cell population responsible for processing innate signals to induce specific CD8^+^ T cell responses, XCR1^+^ DCs are crucial in the generation of successful adaptive immune responses against viral challenges [13,27,76] and the induction of peripheral tolerance [48,77]. More recently, there is mounting evidence that the cross-presenting DC subtype is critical in potentiating anti-tumor responses. Experiments utilizing Batf3^−/−^ mice lacking cross-presenting DCs failed to control syngeneic fibrosarcomas that were rapidly rejected in wildtype mice, with a lack of tumor-specific CTLs implicated [13]. A decrease in tumor-infiltrating CD103^+^ DC due to tumor-intrinsic upregulation of β-catenin resulted in impaired priming and recruitment of effector CD8^+^ T cells into the TME in an induced melanoma model [20,78]. Batf3^−/−^ mice, as generated and characterized by Hildner and colleagues, selectively lack CD8^+^ cDCs and CD103^+^CD11b^neg^ DCs whilst demonstrating equivalent total cell numbers of other DC subtypes [12,13]. In particular, humoral and CD4^+^ T cell responses are not compromised in Bat3^−/−^ mice, nor is CD8^+^ T cell differentiation or ex vivo effector function affected [13]. It may be important in the interpretation of studies utilizing Batf3^−/−^, however, to consider the limitations of this frequently used model. These include as follows: (i) Batf3 is expressed in a number of other cell types including cDC2, albeit not as highly as in cDC1s, (ii) cDC1 depletion is less effective in mice with a C57BL/6 background, compared to other strains such as 129/SvEv, (iii) intracellular infection or IL-12 injection can restore cDC1 development and (iv) regulatory T cells are increased in knock-out mice [13,79,80]. More recently, it has been demonstrated that WDFY4 is required for cross-presentation by cDC1. While WDFY4-deficient mice have normal cDC1 development, anti-tumor immunity is compromised in this strain [27]. Similarly, the XCR1-DTR model allows exclusive depletion of developmentally normal XCR1^+^ DCs following diphtheria toxin injection, although has not yet to our knowledge been used in cancer models [81]. It is anticipated that an increased use of more targeted approaches of cross-presenting DC depletion may overcome the uncertainty associated with the caveats of the Batf3^−/−^ model, unequivocally establishing cross-presenting DCs as an essential component in anti-tumor responses.

In humans, high numbers of cross-presenting BDCA3^+^ DCs in the TME is associated with increased T cell infiltration and improved prognosis in cancer patients [20]. A high CD103^+^/CD103^neg^ DC ratio signature was found to strongly correlate with improved overall patient survival across 12 cancer types [19]. Increased IL-12 expression in human breast cancer patients, a cytokine secreted by activated cross-presenting DC, positively correlated with pathological complete response rates [82]. Despite this, cross-presenting DCs are typically under-represented in both mouse [23,82] and human [19,83] tumors.

### 3.2. XCR1^+^ DCs Potentiate Current Cancer Immunotherapy Strategies

The dependence of several clinically approved immunotherapies on the presence and function of cross-presenting DCs provides significant insight into their crucial role. For example, low numbers of Batf3-dependent DCs in the TME is associated with limited adoptive cell therapy (ACT) success in an inducible mouse model of melanoma [78]. This was explained by an absence of the CD103^+^-derived CXCL9/10 chemokines, resulting in reduced T cell trafficking into the TME [78]. A recent study in mice demonstrated effective sub-therapeutic doses of chimeric antigen receptor (CAR)-T cells against solid tumors when administered with a liposomal RNA vaccine [84]. The RNA-encoded CAR antigen was delivered to all antigen-presenting cells (APCs); however, the increased expansion and effector function of CD8^+^ CAR-T cells implies a vital role for cross-presenting DCs in the established tumor control [84].

Immune checkpoint therapy acts to promote robust CTL responses by activation of costimulatory receptors or blocking of co-inhibitory receptors on tumor-specific T cells. In line with this, the presence and abundance of cDC1 within the TME is associated with improved patient outcomes in several types of cancer [19,20,25,26], and response to checkpoint blockade therapy in mice [20] and humans [25]. Conversely, tumor-bearing Batf3-deficient mice demonstrated non-responsiveness to anti-PD1, anti-PDL1 and anti-CD137 checkpoint blockade [23,85]. B-catenin-mediated depletion of tumor-infiltrating CD103^+^ DCs in mice induced resistance to anti-CTLA4 and anti-PDL1 treatment, which was overcome by the transfer of Flt3L-generated cDC1-like cells stimulated with poly I:C [20]. Direct transfer of pre-activated DCs, or “DC vaccination”, has itself shown promising anti-cancer potential in isolation and in combination with other therapies, which will be discussed in further detail below.

XCR1^+^ DCs can also facilitate anti-cancer immune activity in response to cancer treatments such as radiotherapy, chemotherapy and photodynamic therapy, which are all known to induce immunogenic cell death (ICD) (reviewed in [86,87,88]). ICD is characterized by the release of damage-associated molecular patterns (DAMPs) from dying tumor cells, resulting in the recruitment and activation of immune cells, specifically cross-presenting DCs [89,90]. DAMPs are critical to this process as they promote DC maturation and the provision of the necessary co-stimulatory signals (CD80, CD86, production of type 1 interferon) required for functional activation of T cells, or cross-priming. The distinctive properties of ICD include i) exposure of calreticulin (CRT) and other ER proteins (HSP70, HSP90) at the cell surface, which act as “eat me” signals facilitating antigen uptake by DCs [91,92], ii) secretion of ATP, which promotes recruitment and activation of DCs via interaction with P2RY2 and P2RX7, respectively [93,94], iii) release of annexin A1 (ANXA1) [95,96], which guides DCs to dying tumor cells and binds formyl peptide receptor 1 on DCs to enable uptake of tumor antigens, and, iv) secretion of HMGB1, which interacts with TLR4 on DCs to promote DC maturation and antigen presentation [97]. Accordingly, cross-presenting DCs are key mediators of this process and anti-cancer treatments inducing ICD are impaired in their absence [93,97]. The productive anti-cancer immune response generated by chemotherapy—or radiotherapy—induced ICD is dependent on TLR4-expressing DCs [97]. Similarly, the immunogenic effects of ATP release from dying tumor cells is dependent on IL-β production by DCs and subsequent recruitment of antigen-specific CD8^+^ T cells [93]. Therefore, treatments designed to promote ICD and enhance cross-presenting DC activity have the potential to synergize with both classical cancer treatments and emerging immunotherapy strategies.

### 3.3. The Immunosuppressive TME Inhibits XCR1^+^ DCs, Promoting Immune Evasion

One of the major challenges in cancer treatment is overcoming the immunosuppressive TME. Negative regulation of DC recruitment, differentiation, activation and survival within the TME culminates in defective tumor-specific T cell responses and increased tumor growth [98]. STAT, MAPK, and β-catenin signaling pathways have been implicated in the induction of an immune-suppressed phenotype in tumor-infiltrating DCs [99]. For example, DC hyperphosphorylation of signal transducer and activator of transcription 3 (STAT3), such as in response to pro-inflammatory cytokines, has been associated with poor maturation status and immunosuppression [100,101,102,103]. Tumor-intrinsic upregulation of β-catenin signaling induces down-regulation of the chemoattractants CCL4 [20] and CCL5 [104] in both mice and humans, and is associated with reduced cDC1 and CD8^+^ T cell infiltration, increased tumor growth [20,104], and resistance to anti-PD1 therapy [104]. It has also been demonstrated that β-catenin signaling in DCs can directly inhibit their ability to cross-present, abrogating CD8^+^ T cell-mediated tumor control [105]. DC-expressed TIM3 similarly inhibits cross-presentation by sequestering the alarmin HMGB1 to prevent uptake of immunogenic nucleic acids from dying cancer cells [106].

Secretion of anti-inflammatory cytokines such as IL10 and TGF-β in the TME can impair cDC differentiation and maturation [24,103]. In line with this, DCs isolated from the TME may lack appropriate maturation markers and have a diminished capacity to prime T cells [107]. Tumor cells, macrophages, DCs and Tregs have all been implicated in the production of IL-10 in the TME [108]. Local IL-10 negatively regulates DC maturation and IL-12 production, and consequently, their capacity to cross-prime T cells [109,110]. Tumor-derived CSF-1 recruits macrophages into the TME, resulting in increased IL-10 levels and defective anti-tumor CD8^+^ T cell responses [82]. Tumor-derived MMP-2 has also been found to inhibit IL-12 secretion, and is correlated with poor prognosis in patients [111,112]. Prostaglandin E2 secreted by tumors can also prevent DC recruitment and maturation via inhibition of NK cell function [24]. The loss of Flt3L, CCL5, and XCL1 secretion by tumor-infiltrating NK cells impacts recruitment and survival of cross-presenting DCs, and correlates with increased tumor growth [22,24,25,26] and responsiveness to anti-PD1 therapy [25]. These studies reveal a vital relationship between NK cells and DCs within the TME, and an anti-tumor role for NK cells beyond direct lytic activity [25]. Tumor-derived VEGF subverts NK cell function by blocking Flt3L, profoundly impacting XCR1^+^ DC development and function [113,114,115]. TME-induced stress on DCs can impede antigen cross-presentation and tumor control [116,117]. For example, tumor-derived lipid peroxidation by-products resulted in abnormal lipid accumulation within intratumoral DCs and subsequent dysfunction [115]. This process was mediated by constitutive activation of endoplasmic reticulum (ER) stress response factor XBP1, with knock-out or silencing of XBP1 promoting survival of ovarian cancer-bearing mice [115]. LXRα agonists secreted by tumor cells can similarly promote tumor growth by blocking CCR7-mediated migration of cDCs from the TME to tumor-draining lymph nodes (TDLNs) [118]. One of the major goals of DC-based therapies is to prevent suppression of cross-presenting DC function to enable successful T cell priming and effective anti-tumor responses.

## 4. Targeting Cross-Presenting DC for Cancer Treatment

**Figure 1:**
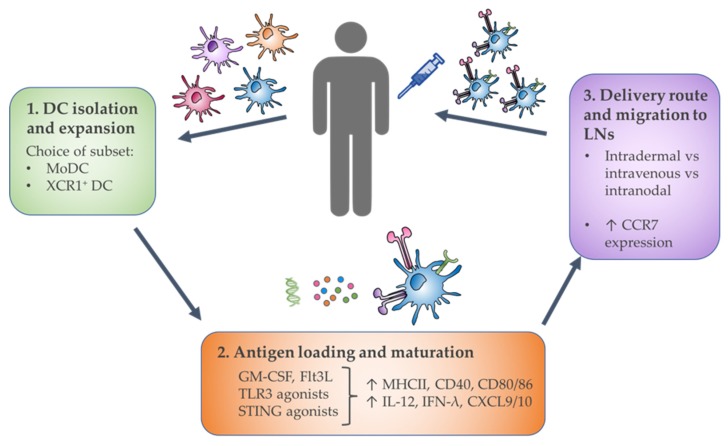
Critical considerations for DC vaccination. DCs may be isolated from patients or generated in vitro. New protocols designed to specifically generate large amounts of XCR1^+^ DCs, rather than MoDCs, may prove beneficial. Ex vivo culture with cytokines and/or TLR/STING agonists upregulates maturation markers on antigen-loaded DCs, and increases cytokine and chemokine secretion. Activated XCR1^+^ DCs are typically injected intradermally, although intranodal improves efficiency of DCs reaching the LNs. Upregulation of CCR7 expression aids in DC migration to the LNs, where they cross-present pre-loaded antigen to naïve CD8^+^ T cells.

**Figure 2:**
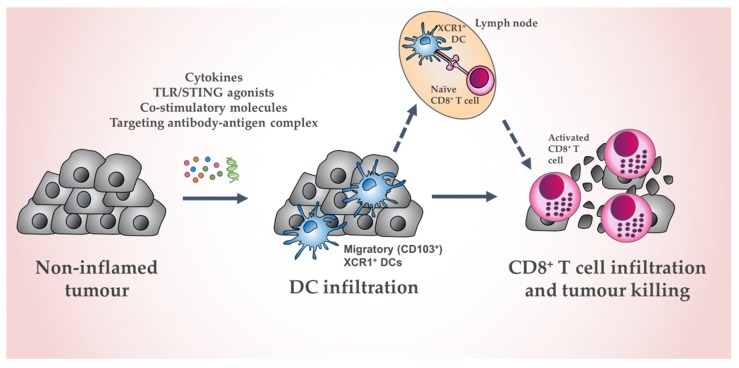
Schematic of in vivo XCR1^+^ DC activation to induce tumor cell death. Administration of cytokines, TLR/STING agonists, co-stimulatory molecules, and targeting antibody–antigen complexes can activate migratory (CD103^+^) XCR1^+^ DC in the tumor and lymphoid-resident XCR1^+^ DC. This can promote antigen acquisition, DC maturation, cross-presentation, and recruitment to the tumor site, culminating in increased CD8^+^ T cell-mediated tumor death.

The unprecedented success of checkpoint blockade therapy has promoted renewed interest in targeting DCs, due to the likelihood of synergy with combined approaches. Indeed, there have been numerous studies establishing the importance of DCs in successful checkpoint blockade response [20,23,25,85,119,120,121]. To date, much of DC-based research and clinical trials utilize monocyte-derived DCs (MoDCs) or broadly target the DC compartment. Given their specialized ability to generate anti-cancer CTL activity and synergize with a number of cancer treatments, the merit in specifically targeting the cross-presenting DC subset in cancer therapy is increasingly being recognized [4,99,122]. One approach involves the exclusive transfer of preloaded, activated cross-presenting DCs as a next-generation DC vaccination protocol [123] (Figure 1). Alternatively, cross-presenting DCs can be targeted in vivo, with the administration of adjuvants, chemokines, and targeting strategies designed to activate, recruit and deliver antigen to cross-presenting DCs (summarized in Table 2 and Figure 2).

### 4.1. DC Vaccination

DC vaccination can involve the transfer of pre-activated DCs loaded ex vivo with antigen to generate T cell responses. The last thirty years have produced a prolific number of clinical trials in the field of anti-cancer DC vaccination, albeit success has been limited [123,124]. Sipuleucel-T (Provenge) is the only DC-based cancer vaccine that is FDA-approved for clinical use against castration-resistant prostate cancer [98]. It consists of autologous blood-derived APCs loaded with prostatic acid phosphatase and GM-CSF fusion protein [5]. Whilst clearly a promising success story, with median overall survival prolonged by 4.1 months, advances are necessary for wider use and improved durable responses [6]. With increased understanding of DC subtype specialties and the limitations underlying current vaccination strategies, the opportunity exists to develop improved next-generation DC vaccines for greater clinical benefit.

One promising approach is to specifically utilize the cross-presenting DC subtype for vaccination. We know that XCR1^+^ cross-presenting DCs are far more effective at generating CTL responses than other DC subtypes [59,69]. Despite this, the majority of clinical trials for DC vaccination to date have utilized MoDCs derived from patient blood monocytes or CD34^+^ precursors [124,125,126]. This is partly due to the lack of knowledge regarding functional specialties of the various DC subtypes, particularly for early vaccination attempts, but also because of the ability to readily generate substantial quantities of MoDCs [126]. There is mounting evidence, however, that these are not the optimal DC subtype to produce successful anti-cancer immune responses [127]. MoDCs do not functionally reflect in vivo cross-presenting DCs, and possess significantly poorer antigen presentation capabilities [10,128]. Studies in mice have demonstrated improved efficacy with the use of a cross-presenting DC vaccine, relative to an equivalent MoDC approach [120]. Unfortunately, the current difficulty in obtaining sufficient quantities of cross-presenting DC from patient blood limits the translation of this approach to humans. It is hoped that the advent of new differentiation protocols will allow the use of this more targeted approach to generate CTL responses in humans in the future [51,127,129]. Indeed, there have been a number of recent advances in the large-scale generation of cross-presenting XCR1^+^ DCs in vitro using novel cell culture methods [130] and protocols employing CD34^+^ stem cells [131,132].

Further opportunities to improve DC vaccination strategies include the addition of appropriate adjuvants to boost DC function, and methods to encourage migration into the TDLNs. It has been shown that autologous DCs are often dysfunctional, inducing T cell tolerance rather than activation due to a lack of maturation markers [107,123]. However, this can be overcome with appropriate adjuvant stimulation [107,130]. Importantly, it is necessary to consider the efficacy of specific adjuvants that directly target cross-presenting DCs. The various DC subtypes display differential responsiveness to specific adjuvants, particularly TLR agonists, such that those promoting MoDC activation do not affect XCR1^+^ DC function in a similar manner [4]. One of the primary difficulties faced in DC vaccination approaches is ensuring successful migration to the TDLNs, which is typically poor [133]. Most DC vaccines are administered intradermally, in close proximity to superficial LNs, and yet only 5% of DCs traffic to the LNs using this vaccination route [123,134]. One strategy to help augment this is ultrasound-guided intranodal injection; however, this is technically challenging [123]. A phase I trial demonstrated superior T cell function with intranodal injection of an autologous peptide-pulsed DC vaccine, as compared to intravenous or intradermal injection routes [135]. Developing protocols to promote appropriate chemokine receptor expression may provide an alternate approach to optimize DC trafficking to the LNs.

Overall, autologous DC vaccination has several putative advantages when compared to targeting DCs in vivo. Activating DCs ex vivo bypasses potential tolerogenic signals present in the body, particularly within the immunosuppressive TME [5]. It also circumvents the dependence on successful transportation of antigens and adjuvants to the necessary location and targeted cells, which can be relatively inefficient [122]. Moreover, it allows for quality control prior to transfer to ensure the appropriate activation and function of the transferred DCs [5]. Conversely, there are also arguments favoring the targeting of DCs in vivo. Autologous DC vaccination requires the large-scale generation of DCs ex vivo, which can be time-consuming, complex and expensive [122]. It is also reliant on the successful trafficking of DCs to the TDLNs for T cell priming [133]. Delivery of antigen and stimulatory adjuvants in vivo may ultimately mitigate these limitations, albeit at the expense of other restrictions.

**Table 2:** Agents promoting cross-presenting DC development, maturation, function, and/or migration.

			THERAPEUTIC EXAMPLES	
AGENT		RESPONSE	Murine	Clinical Trials in Cancer
**CYTOKINES**	GM-CSF (e.g., GVAX, T-VEC)	Differentiation, mobilization and activation of cDCs	[136,137,138]	NCT00065442—FDA approved (Sipuleucel-T) NCT00769704—FDA approved (T-VEC) NCT01435499 NCT01740297
	Flt3L (e.g., CDX-301)	Differentiation, mobilization and expansion of XCR1^+^ DCs	[23,121,139]	NCT01465139 [140] NCT02129075 NCT02839265 NCT01976585
	Type I IFN	Upregulation of DC maturation markers (e.g., MHCII, CD40, CD80/86)	[119,141,142,143,144,145]	NCT00006249—FDA approved (Pegylated IFNα2) NCT00204529 NCT01545141
**TLR AGONISTS**	TLR3 agonists (e.g., poly I:C and its derivatives)		[23,85,121,146]	NCT01188096 [134] NCT01734564 NCT02129075
**STING AGONISTS**	Cyclic dinucleotides (e.g., ADU-S100)		[147,148,149]	NCT02675439 NCT03172936
	Non-nucleotidic STING agonists (e.g., DMXAA)		[150]	NCT00662597 [151]
**CO-STIMULATORY MOLECULES**	CD40L/anti-CD40 antibodies	Provide co-stimulation during T cell priming	[152,153,154,155]	NCT00458679 NCT02482168 NCT00678119
**INHIBITORS**	STAT3 inhibitors (e.g., OPB-51602)	Prevent immunosuppression to promote DC maturation	[100,101]	NCT00955812 NCT01423903
	IDO inhibitors (e.g., 1-MT, NLG919)		[156,157]	NCT01042535 NCT01792050
**CHEMOKINES**	CCL5	Recruitment of cDCs	[158]	Nil.
	XCL1	Recruitment of XCR1^+^ DCs	[26,159]	Nil.

### 4.2. Adjuvants

DC maturation status and upregulation of co-stimulatory receptors is critical for successful T cell priming, yet this is often undermined by a lack of appropriate stimulatory signals and/or an immunosuppressive TME [160]. Adjuvants such as cytokines, toll-like receptor (TLR) and stimulator of interferon genes (STING) agonists, or co-stimulatory receptor ligands aim to overcome this by promoting DC activation, proliferation, and recruitment. They can be used to stimulate DC in vivo or during the generation of DCs ex vivo for DC vaccination [161]. This is typically demonstrated by an increase in proliferation, upregulation of MHC complexes and co-stimulatory molecules (e.g., CD40, CD80, CD86), cytokine secretion (e.g., IL-12, IFN) and chemokine expression (e.g., CCR7).

The cytokines GM-CSF and Flt3L both act to stimulate DC development and differentiation. GM-CSF has been used therapeutically for many years, and is commonly used in conjunction with DC vaccines and other cancer immunotherapies to support tumor rejection [138,162,163]. GVAX refers to a vaccine platform utilizing irradiated tumor cells engineered to express GM-CSF [163]. Widely used, it has been demonstrated to induce tumor-specific T cell responses, synergizing with PD1 and CTLA4 blockade in pre-clinical models of pancreatic and prostate cancer, respectively (reviewed in [28]). Clinical trials testing the efficacy of GVAX combined with anti-PD1 therapy and/or immunogenic chemotherapy are currently underway in patients with advanced pancreatic and colon cancer (NCT02648282, NCT03153410, NCT03006302, NCT03767582, NCT01966289). However, to better target the cross-presenting DC population, Flt3L may be a more appropriate cytokine. GM-CSF acts to broadly induce differentiation of a number of DC subsets, particularly inflammatory DCs [69,164]. In contrast, whilst Flt3L does promote expansion of a number of DC subtypes, it is the limiting cytokine for the development of XCR1^+^ DCs in both mice and men [69,165,166]. In vivo injection of Flt3L dramatically boosts CD8^+^XCR1^+^ and CD103^+^XCR1^+^ DC numbers, establishing them as the dominant DC population in the spleen [165,167,168] and tumor [23], respectively. Flt3L-induced expansion of CD8α^+^ DCs induces expansion of antigen-specific CD8^+^ T cells, and mediates tumor regression in synergy with TLR agonists and checkpoint blockade [23,121,139,152,169]. Curran et al. compared whole cell vaccination with GM-CSF- and Flt3L-expressing tumor cells, and found significantly higher DC and CD8^+^ T cell infiltration with the Flt3L treatment [139]. The preference for Flt3L to enhance cross-presenting DCs may be beneficial in the treatment of cancer, particularly when used in combination with other therapies targeting this subset. In line with this, clinical trials testing the efficacy of recombinant human Flt3L (CDX-301, Celldex Therapeutics) in various combinations with anti-CD40 (CDX-1401), poly-ICLC, pembrolizumab and radiotherapy are currently underway for patients with melanoma (NCT02129075), lymphoma (NCT01976585), prostate cancer (NCT03835533) and other advanced malignancies (NCT03329950, NCT03789097).

TLR and STING agonists can act on DCs to promote maturation and cytokine secretion, thus mediating T cell activation and effector function [170]. Of the TLR agonists, those targeting TLR3 are likely to be the most appropriate choice for effective activation of cross-presenting DCs. Despite wide-spread TLR expression on mouse XCR1^+^ DCs, human XCR1^+^ DCs exclusively express TLR3 (see Table 2). TLR3 agonists, such as poly I:C, are shown to synergize with both checkpoint blockade [23] and DC vaccination [171], by activating DCs and promoting cross-presentation to produce enhanced tumor control [23,85,121,171]. Hammerich et al. demonstrated anti-tumor efficacy with a combined in situ vaccination approach, whereby mice or patients (NCT01976585) received a TLR3 agonist, Flt3L, and radiotherapy [121]. A requirement for Batf3-dependent cDC1s in the anti-cancer effects of poly I:C has been demonstrated in this and other studies [51,121,172]. Binding of TLR3 by double-stranded RNA results in activation of the NF-kB pathway and the transcription factor interferon regulating factor 3 (IRF3), to ultimately induce the production of pro-inflammatory cytokines, particularly the type I interferons (IFNs) [173,174]. Type I IFN signaling has been shown to promote tumor rejection in a process mediated by CD8^+^ DCs in the TDLNs [119,141,142]. The enhancement of cross-presentation by type I IFN has been implicated previously [143,144,145]. It should be noted that TLR9 agonists have also been shown to activate human cDCs to promote cancer-specific CD8^+^ T cell responses [175]. Similar to TLR3 agonists, STING agonists also act to induce secretion of type I IFN, and other inflammatory cytokines, via NF-kB and IRF3 [176,177,178]. Activation of the STING pathway in vitro induces DC activation, with impaired CD8^+^ T cell priming in mice lacking this pathway [147]. Direct intratumoral injection of STING agonists was found to mediate regression of both primary and metastatic tumor lesions, in a process requiring Batf3-dependent DCs [148]. Clearly activation of cross-presenting DCs with TLR and STING agonists, or their downstream cytokines, has great implications for cancer immunotherapy.

Engagement of CD40 on DCs, typically via interaction with CD40L on helper CD4^+^ T cells, is essential for successful T cell priming [161,179]. Promising studies in both mice and humans indicate delivery of agonistic antibodies to CD40 activates DCs both in vivo and ex vivo to induce tumor-specific CD8^+^ T cell responses [152,153,155], and synergize with poly I:C treatment [180] independent of CD4^+^ T cell help. However, therapeutic use is limited due to severe toxicities associated with anti-CD40 treatment in the clinic, primarily cytokine release syndrome [155]. These side-effects are being addressed by the development of slow-release formulations, and targeted delivery to the TDLNs [154]. In summary, the use of adjuvants which induce DC maturation and activation are likely to be critical for the effective use of DC-based immunotherapies against cancer.

### 4.3. Chemokines

Chemokines and their receptors are vital in anti-cancer immune responses for their ability to direct the trafficking of DCs and other immune cells. For example, CCR7 expression on migratory CD103^+^ DCs that have encountered antigen facilitates their migration from the TME to tumor-draining LNs for the cross-presentation of antigen to CD8^+^ T cells [23,29]. CCR7, in addition to NK cell-secreted CCL5 and XCL1, may also be involved in the initial recruitment of DCs into the TME [26,118,181,182]. The use of XCL1 is of particular interest due to its ability to specifically attract cross-presenting XCR1^+^ DCs. This has been exploited in the context of transgene therapy, whereby local expression of XCL1 and the differentiation factor Flt3L was induced by intratumoral injection of a Semliki Forest Virus (SFV)-based vector encoding these genes [183]. Treatment was associated with an increase in intratumoral CD103^+^ cDC1s, enhanced cross-priming, and a Batf3-dependent delay in tumor progression [183]. Importantly, expression of both Flt3L and XCL1 was required for optimal anti-tumor effects [183]. In addition to the chemoattractant and differentiation/survival properties of XCL1 and Flt3L respectively, the SFV vector itself releases viral dsRNA, inducing XCR1^+^ DC maturation via TLR3 in an elegant synergy of XCR1^+^ DC-targeting agents [183,184]. Once recruited to the TME, DCs secrete chemokines that aid in T cell trafficking into tumors. For example, CD103^+^ DC-derived CXCL9/10 promotes CD8^+^ T cell infiltration into the TME [78]. This CXCL9/10 secretion by CD103^+^ DCs was found to be essential for response to anti-PD1 treatment [185]. Consideration of chemokines and their receptors is likely to be imperative in overcoming the substantial limitations presented with current DC-based vaccination strategies.

### 4.4. Targeting Antibodies

A significant challenge for in vivo targeting of DCs is the delivery of antigen and/or adjuvant to DCs in the lymph node and/or tumor site. Complexing antigen with novel targeting antibodies is providing a promising solution to efficiently direct antigen to these DCs, with antibody-adjuvant complexes likely to reduce dose-limiting toxicities [5]. Antibodies are commonly directed against C-type lectin receptors (CLRs), due to their presence on the surface of DCs, and involvement in endocytosis [186]. Anti-DEC205-coupled antigens demonstrate a greater likelihood of cross-presentation relative to non-conjugated antigen [187,188], resulting in greater CD8^+^ T cell activation [32]. Limited clinical responses were observed during a phase I clinical trial using this targeting strategy in combination with resiquimod and/or poly-ICLC for the treatment of oesophageal squamous cell carcinoma [33]. Targeting of CD40 has also been investigated, with the simultaneous activation of DCs promoting successful cross-presentation [189,190].

Of particular interest are antibodies which enable the exclusive targeting of antigen and/or adjuvant to cross-presenting XCR1^+^ DCs, due to the subtype-specific expression of their targets. CLEC9A-targeting antibodies bound to tumor-associated antigens have been shown to induce potent CD8^+^ T cell responses [70,191], which can lead to effective tumor control in mice [31]. Targeting of antigen to the XCR1 receptor allows even greater specificity for the cross-presenting DC subset [192]. Early studies demonstrate enhanced efficiencies of XCL1-bound antigen to induce CD8^+^ T cell priming compared to untargeted antigen [193], with the potential to modify XCL1 for increased chemotactic effect [159]. Recent studies have demonstrated that gene [194] and protein [159,193,195] antigen-XCL1 fusion complexes can increase antigen-specific CD8^+^ T cell priming and infiltration into the tumor relative to untargeted antigen, with the potential to modify XCL1 for increased chemotactic effect [159]. This was associated with improved tumor control [159,193,194,195] and could synergize with anti-PD1 treatment [194]. Importantly for translation, Chen et al. demonstrated a human XCL1-Glypican-3 fusion gene immunization protocol could target human XCR1^+^ DCs both in vitro and in vivo, and induce anti-HCC effects in a HCC-PDC model [194]. The identification of XCR1 as a defining marker of cross-presenting DCs remains relatively recent, and so further advances in our understanding of this subset may reveal additional putative targets [15,16]. For example, unveiling of XCR1^+^ DC precursors could present targets that will increase the longevity of antigen presentation.

## 5. Conclusions

Substantial investigation into the basic biology and therapeutic potential of DCs has yielded numerous clinical trials, and yet success thus far has been limited. With the remarkable success of checkpoint blockade, there has been a resurgence of interest in DC-based strategies due to the likelihood of synergy with a combined approach. Here, we have discussed the XCR1^+^ cross-presenting subset as a potential focus of next-generation DC therapies, due to their specialized ability to prime effector CD8^+^ T cells and role in mediating anti-tumor responses. Careful consideration on the most effective interventions to enhance migration, activation, maturation and function of XCR1^+^ DCs is likely to be vital to unleash their full anti-cancer potential.

## Figures and Tables

**Table 1:** Phenotype of lymphoid-resident and migratory cross-presenting dendritic cells (DCs) in mice and humans.

	MOUSE		HUMAN	
	Lymphoid-Resident	Migratory	Lymphoid-Resident	Migratory
**SURFACE MARKERS**	CD11c, MHCII, **CD8**, **XCR1**, CLEC9A, DEC205, Necl2	CD11c^low^, MHCII, **CD103**, langerin, **XCR1**, CLEC9A, DEC205	CD11c^low^, HLA-DR, **CD141** **(BDCA-3)**, **XCR1**, CLEC9A, DEC205, Necl2	CD11c^low^, HLA-DR, **CD141^hi^** **(BDCA-3)**, **XCR1**, CLEC9A, Necl2, CD103 ^a^
DEVELOPMENTAL TF * & CYTOKINES	**Batf3**, IRF8, Id2, Bcl6, NFIL3, Flt3L	**Batf3**, IRF8, Id2, Bcl6, Flt3L, GM-CSF	**Batf3**, IRF8, Id2, Bcl6, FLt3L	**Batf3**, IRF8, Id2, Bcl6
**PRR EXPRESSION**	**TLR-3**, 4, 9, 11, 12, 13, STING, CLEC12A	TLR-1, 3, 6, 8, 11, 12, STING, CLEC12A	**TLR-3**, 8, 10, STING, CLEC12A	**TLR-3**
**CYTOKINES & CHEMOKINES**	**IL-12**, IFN-λ, CXCL9/10	**IL-12**, CXCL9/10	IL-12 (low), type I IFN, IFN-λ, CXCL9/10	TNFα, CXCL10

^a^ Only present on intestinal CD141^hi^ cells, with only the SIRPα^neg^ as cross-presenters. * TF = transcription factors. Subset-defining features are in bold. References are located throughout the text.
